# Supplementary material for: Allium sativum Extract Chemical Composition, Antioxidant Activity and Antifungal Effect against Meyerozyma guilliermondii and Rhodotorula mucilaginosa Causing Onychomycosis
Source: Molecules. 2019 Oct 31;24(21):3958. doi: 10.3390/molecules24213958 (PMC6865177; doi:10.3390/molecules24213958)
Supplement: Supplementary file 1 [file molecules-24-03958-s001.pdf]

Allium sativum extract chemical composition, antioxidant activity and antifungal effect against *Meyerozyma guilliermondii* and *Rhodotorula mucilaginosa* causing onychomycosis

Marcel Pârvu [1*], Cătălin A. Moţ [2], Alina E. Pârvu [3], Cristina Mircea [1], Leander Stoeber [4], Oana Roşca-Casian [5], Adrian B. Ţigu[1,6]

Results

Phytochemical analysis of garlic extract

In the analysed garlic extract the concentration of alliin was 1410 μg/mL and of allicin was 380 μg/mL (Figure S1, Table S1). Allicin presents a distinct spectral shoulder around 250 nm due to the thiosulfinate group. Mass spectra of the separated compounds were also monitored, in the positive mode (Figure S1A). Alliin exhibit an intense molecular peak at the expected 177.2 m/z (M^+) while allicin beside the molecular peak at 162.2 m/z (M^+) showed another ion, 120.2 m/z due to an allyl group loss and another peak at 184.2 m/z which is most probably an adduct with sodium ion (MNa^+).

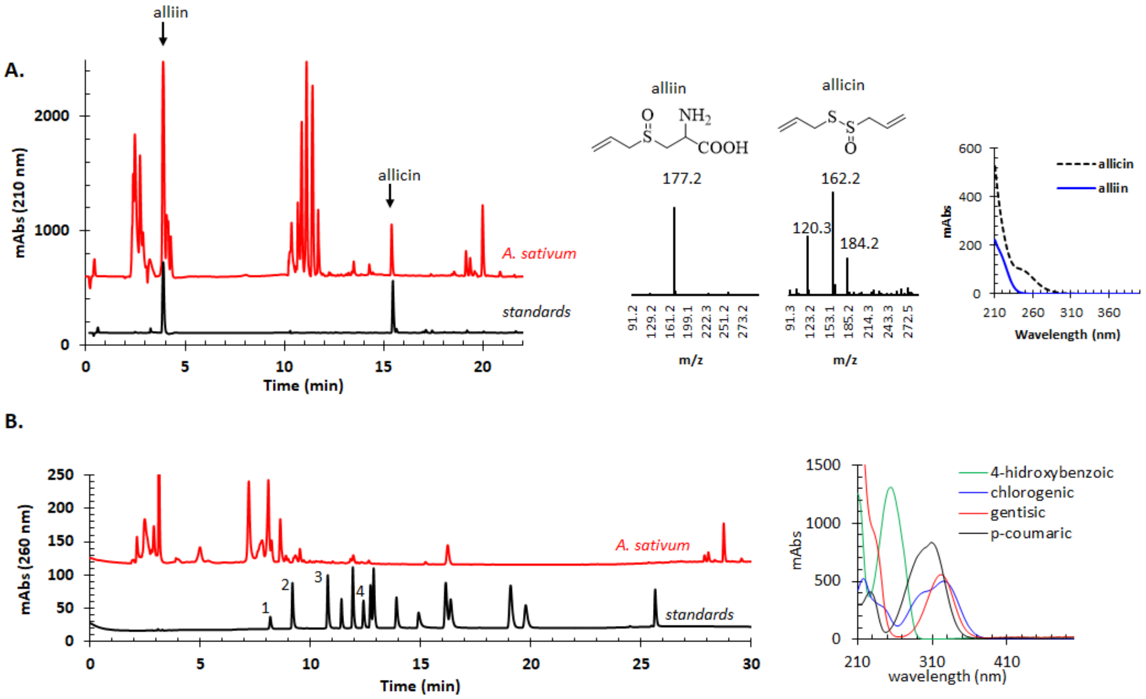

Figure S1. **A.** Chromatographic separation (210 nm detection) of the *A. sativum* extract for alliin and allicin determination and the MS and UV-vis absorption molecular spectra of the two separated compounds. **B.** Chromatographic separation (260 nm detection) of the *A. sativum* extract for phenolics determination and the UV-vis absorption molecular spectra of the four determined compounds (1 - gentisic acid, 2 - chlorogenic acid, 3 - 4-hydroxybenzoic acid, 6 - p-coumaric acid).

Table S1. Elution time, analytical method characteristics and determined concentrations of alliin and allicin in the studied *A. sativum* extract (n=4).

No	Compounds	$t_{elution}$ (min)	R^2	LOD (μg/g)	*A. sativum* (μg/g)

1	Alliin	3.77	0.9999	5.8	1410±50
2	Allicin	15.40	0.9999	14.1	380±15

LOD – limit of detection, R^2 – coefficient of determination for the calibration curves (at six levels of concentrations). Indicated intervals represents the average ± standard deviations (n = 4).

Regarding phenolics, only gentisic acid, chlorogenic acid, 4-hydroxybenzoic acid, and p-coumaric acid were found to be above the limit of quantification (LOQ) (Figure S1B, Table S2), but no flavonoid was detected.

Table 2. Elution time, analytical method characteristics and determined concentrations of phenolic acids and flavonoids in the studied *A. sativum* extract (n=4).

No	Compounds	$t_{elution}$ (min)	R^2	LOD (μg/g)	*A. sativum* (μg/g)
1	Gentisic acid	8.13	0.9997	3.4	60±5
2	Chlorogenic acid	9.15	0.9995	4.6	65±5
3	4-hydroxybenzoic	10.31	0.9999	2.3	25±3
4	p-coumaric acid	12.38	0.9999	1.9	44±4

LOD – limit of detection, R^2 – coefficient of determination for the calibration curves (at six levels of concentrations). Indicated intervals represents the average ± standard deviations (n = 4).

Chromatograms of the analysed extract exhibit other important peaks that were not identified by mass alone. The UV spectra of the unidentified peaks, as registered by DAD detector after HPLC separation, and the scatterplot of the scores for the first two components when PCA was applied on these spectra, are illustrated in Figure S2A and S2B respectively. The data indicate that these main unidentified constituents corresponding to the chromatographic peaks of the *A. sativum* extract are nonphenolic in nature and rather similar with alliin and allicin in terms of UV-vis spectral features, most probably non-aromatic sulphur-containing compounds such as peptides and their derivatives.

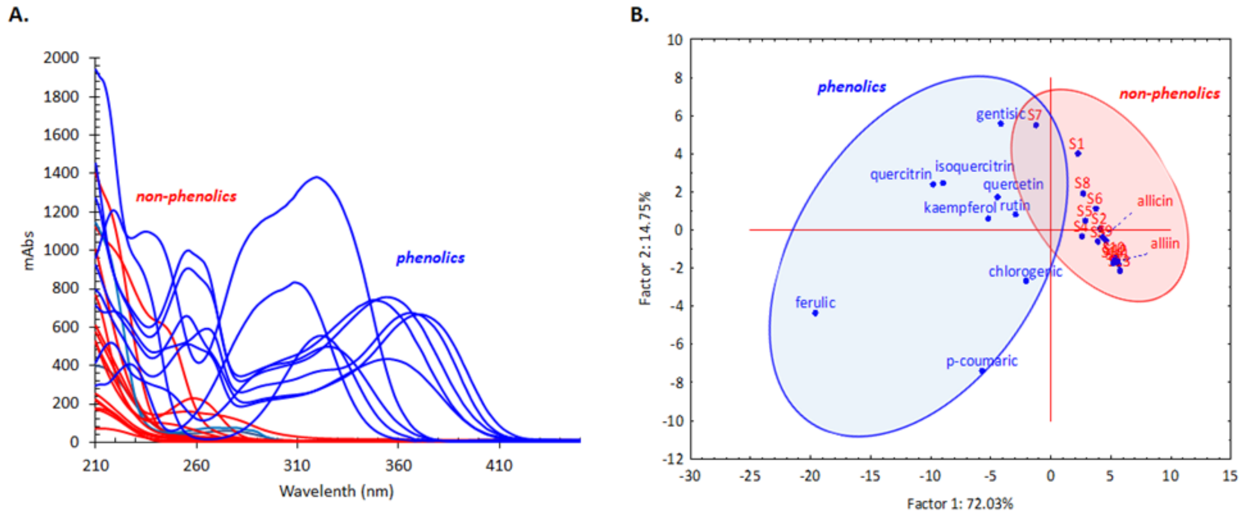

Figure S2. A. Mean UV-vis absorption molecular spectra of *A. sativum* main chromatographic peaks and of the standards, indicating the two classes, phenolics and non-phenolics compounds. **B.** Scatterplot of the first two components in the Principal Component Analysis (PCA) applied on the UV-vis spectra. Standards are indicated by name while the main chromatographic peaks are labelled with S1-S14.

Chromatograms of the analysed extract exhibit other important peaks that were not identified by mass alone. The UV spectra of the unidentified peaks, as registered by DAD detector after HPLC separation, and

the scatterplot of the scores for the first two components when PCA was applied on these spectra, are illustrated in Figure 2A and 2B respectively. The data indicate that these main unidentified constituents corresponding to the chromatographic peaks of the *A. sativum* extract are nonphenolic in nature and rather similar with alliin and allicin in terms of UV-vis spectral features, most probably non-aromatic sulphur-containing compounds such as peptides and their derivatives.

Materials and Methods

Phytochemical analysis

The separations were performed on an Agilent 1200 HPLC system (Waldbronn, Germany) equipped with an on-line vacuum degasser, quaternary pump, temperature-controlled sample tray, automatic injector, a column thermostat compartment a DAD detector followed by a 6320 Ion Trap MS detector. The chromatographic separations were run on a Zorbax SB-C18 column (250 mm x 4.6 mm, 5 μm particle size) also from Agilent. The injection volume was 15 μL (0.22 μm filtered extract), the column temperature was set to 30 °C and the flow rate was 1 mL/min. Several preliminary tests were employed for method optimization using different experimental conditions. The optimum methods consisted of a multistep gradient elution system using solvent A, 10 mM ammonium formiate pH 2.5 for protocol one and ammonium acetate pH 5.5 for second protocol and as solvent B acetonitrile. For the first protocol, the steps were as follows: 0-5 min isocratic at 0% B, 5-14 min from 0 to 70% B, 14-15 min from 70% to 90% B, 15-18 min from 90% to 100% B, 18-22 min isocratic at 100% B and 22-22.1 min back to 0% B where was kept until 25 min.

For the second protocol, the steps were as follows: 0-2 min isocratic at 5% B, 2-10 min from 5 to 35% B, 10-20 min from 35% to 45% B, 20-25 min from 45% to 95% B, 25-28 min from 95% to 100% B, 28-32 min isocratic at 100% B and 32-32.1 min back to 5% B where was kept until 35 min. The UV-Vis detection of the compounds was performed using the DAD detector that measured the entire spectrum in 210-600 nm region (2 nm resolution), every 2 seconds and the chromatograms were monitored at 210, 220, 230, 240 and 280 nm for first protocol and 242, 260, 280, 320 and 340 for second protocol. As standards there were allicin, alliin, gentisic acid, 3,4-dihydroxybenzoic acid, chlorogenic acid, 4-hydroxybenzoic acid, cafeic acid, syringic acid, rutin, isoquercitrin, p-coumaric acid, quercitrin, ferulic acid, myricetin, morin, luteolin, quercetin, apigenin, kaempferol, galangin, all of analytical grade purity for different commercial available sources, except allicin which was synthesized as described below. Calibration curve was constructed 39, 78, 156, 313, 625, 1250 μg/mL for allicin and alliin determination, in the case of first protocol and 35, 53, 70, 105, 140, 210, 280 μg/mL for phenolic compounds, using the area of the peak by integration employed by the Agilent soft. The limit of detection (LOD) and limit of quantification (LOQ) were determined by formula LOD = (3.3 × standard deviations of intercept)/calibration curve slope and LOQ = (10 × standard deviation of intercept)/calibration curve slope, respectively. The identification of the compounds was employed by both chromatographic retention time and spectral similarities that were done by the built-in soft, as well as using the MS spectrum. The chromatograms and the mean spectra of the main chromatographic peaks were exported and analysed using Excel and Statistica 10 software packages.

In order to investigate the chemical nature of these compounds, a chemometric approach of chemo-mapping was applied on their UV-vis spectra. For this purpose, the UV-vis spectra of the main chromatographic peaks (with a maximum higher than 50 mAbs at 210 nm, 14 compounds, the four detected phenolic compounds were not among them) and the standards used in the calibrations were exported from the Agilent ChemStation soft and were analysed with Principal Component Analysis (PCA) using Statistica 10.

Allicin synthesis and characterization

Allicin was synthesized and analyzed according to Jansen *et al*., 1987 [1]. Briefly, 200 µL pure diallyldisulfide (Sigma Aldrich, St. Louis, USA) was dissolved in 14 mL dichloromethane to which 6 mL solution containing 357.7 mg 3-chloroperbenzoic (Sigma-Adrich, St. Louis, USA) was added dropwise under vigorous stirring in a cooling bath at -10 °C during one hour. Following this, the resulting solution was further kept at room temperature for one hour and washed two times with 2.5% sodium bicarbonate and water and then the organic phase was treated with anhydrous sodium sulfate for water removal. The solvent was removed under vacuum at room temperature and the resulting clear, oily and garlic-smelling substance was aliquoted and kept at -80 °C. MS and ^{1}H, ^{13}C NMR analysis proved it was allicin at purity higher than 96%. ^{1}H-NMR (400 MHz, CDCl3): δ3.70–3.85 (m, 4, J = 4.74 Hz, =CH-CH$_2$-S-S(=O) and =CH-CH$_2$-S(=O)-); 5.16–5.40 (m, 2, J = 6.64 Hz, =CH-CH$_2$-S-S(=O) and =CH-CH$_2$-S(=O)-); 5.80–5.90 (m, 4, J = 1.64 Hz, CH$_2$=CH-CH$_2$-S-S(=O)-S- and CH$_2$=CH-CH$_2$-S(=O)-); 13C-NMR: (125 MHz, CDCl3) δ 35.04, 59.78, 119.05, 124.05, 125.82, 132.91.

Reference

1. Jansen, H.; Müller, B.; Knobloch, K., Allicin characterization and its determination by HPLC. *Planta med* **1987,** *53*, 559-562, doi: 10.1055/s-2006-962811.
